# Use of Internet Viral Marketing to Promote Smoke-Free Lifestyles among Chinese Adolescents

**DOI:** 10.1371/journal.pone.0099082

**Published:** 2014-06-09

**Authors:** Patrick Ip, Tai-Hing Lam, Sophia Siu-Chee Chan, Frederick Ka-Wing Ho, Lewis A. Lo, Ivy Wing-Sze Chiu, Wilfred Hing-Sang Wong, Chun-Bong Chow

**Affiliations:** 1 Department of Paediatrics and Adolescent Medicine, The University of Hong Kong, Hong Kong Special Administrative Region, China; 2 School of Public Health, The University of Hong Kong, Hong Kong Special Administrative Region, China; 3 Food and Health Bureau, Government of the Hong Kong Special Administrative Region, Hong Kong Special Administrative Region, China; The University of Auckland, New Zealand

## Abstract

**Purpose:**

Youth smoking is a global public health concern. Health educators are increasingly using Internet-based technologies, but the effectiveness of Internet viral marketing in promoting health remains uncertain. This prospective pilot study assessed the efficacy of an online game-based viral marketing campaign in promoting a smoke-free attitude among Chinese adolescents.

**Methods:**

One hundred and twenty-one Hong Kong Chinese adolescents aged 10 to 24 were invited to participate in an online multiple-choice quiz game competition designed to deliver tobacco-related health information. Participants were encouraged to refer others to join. A zero-inflated negative binomial model was used to explore the factors contributing to the referral process. Latent transition analysis utilising a pre- and post-game survey was used to detect attitudinal changes toward smoking.

**Results:**

The number of participants increased almost eightfold from 121 to 928 (34.6% current or ex-smokers) during the 22-day campaign. Participants exhibited significant attitudinal change, with 73% holding negative attitudes toward smoking after the campaign compared to 57% before it. The transition probabilities from positive to negative and neutral to negative attitudes were 0.52 and 0.48, respectively. It was also found that attempting every 20 quiz questions was associated with lower perceived smoking decision in future (OR  = 0.95, p-value <0.01).

**Conclusions:**

Our online game-based viral marketing programme was effective in reaching a large number of smoking and non-smoking participants and changing their attitudes toward smoking. It constitutes a promising practical and cost-effective model for engaging young smokers and promulgating smoking-related health information among Chinese adolescents.

## Introduction

Adolescence is a period of increased vulnerability to smoking initiation.[1], [2] In Hong Kong, 67.1% of daily cigarette smokers report beginning to smoke weekly between the ages of 10 and 19, and another 29.0% between at 20 to 29.[3] From 2009 to 2011, the daily smoking rate has risen 6% for male aged between 15 and 19 and 8% for female in the same age group.[4], [5] These are alarming because early smoking initiation has stronger physical and behavioural consequences. For example, smoking during adolescence and young adulthood impairs lung growth and accelerates the onset of lung function decline in addition to causing many cardiovascular diseases and cancers.[6] Early initiation of tobacco use is also associated with other risk behaviours such as alcohol use and early sexual intercourse, which together associated with substance abuse and certain mental disorders in adulthood.[7], [8] Preventing smoking initiation and encouraging smoking cessation during adolescence is a real challenge for health professionals.

Both ‘upstream’ and ‘downstream’ interventions have been developed to tackle this worrying trend. Policy interventions for tobacco control, such as tobacco tax increase and smoking ban, were perceived to be highly effective and impose limited side-effect to country economy.[9] However, this approach is not without drawbacks: increased taxation was argued to be ethically unjust as some smokers may not be fully autonomous for smoking decision[10] and there is evidence suggesting that indoor smoking ban may increase the second hand smoking exposure of children [11].

The limitations of ‘upstream’ policy interventions could actually be supplemented by ‘downstream’ individualised strategies, such as health education, attitudinal and behavioural interventions.[11] Health promotion through traditional media channels such as television, radio, and newspapers is well established,[12], [13] yet today's adolescents are increasingly inclined to search for health information online.[14] A local study found that 98% of the 405 subjects aged 15 to 24 cited Internet as their chief source of information, 43% citing it as their preferred health education source, compared to 27% for television, 17% for newspapers, and 11% for magazines.[15] The Internet is thus increasingly recognised as a practical and cost-effective platform for health information delivery[16].

Internet viral marketing, or the electronic ‘word-of-mouth’ dissemination of information, is one of the best-recognised forms of Internet-based marketing, and accordingly has received considerable attention in the commercial arena in recent years [17]–[19]. Discussions have suggested viral marketing has a lot of potential when compared to traditional media, especially in its message propagation speed and ability in audience reaching[20].

Viral marketing may be especially applicable to anti-smoking campaigns aimed at adolescents and young adults, as peer influence is known to be one of the most powerful factors in their decision to start smoking and their smoking patterns [21]–[24]. Of the 657,000 daily smokers in Hong Kong in 2011, 61.0% reported that they had begun smoking because of the ‘influence of friends’ [25]. Studies have also noted that adolescents are likely to adopt similar smoking behaviour to their friends over time [22]. Among smoking adolescents, having friends who smoke is associated with greater nicotine dependence [26]. Moreover, adolescent smokers' intention to quit is also affected by the smoking status of their close friends [24].

Despite the demonstrated success of viral marketing in the commercial and other arenas, evidence of utilising Internet viral marketing in health education and intervention to achieve attitudinal and/or behavioural change is still limited [27]–[30]. In fact, only 10 viral marketing interventions met the inclusion criteria (e.g. evaluable behavioural outcome and a reference for comparison) in a recent systematic review which searched through six independent databases [30]. Knowledge on the reach of Internet viral marketing, its effects on individuals' behaviour, attitudes and health improvements, and the determinants of the person-to-person information exchange process remains elusive. Thus, in late 2012, we conducted a pilot study on a viral marketing campaign targeting adolescents and young adults in Hong Kong. We set up an online quiz game to deliver tobacco-related information and participants were encouraged to refer others to the game through online platforms.

Our aim in this prospective study is, therefore, to reach a large number of young people (both smokers and non-smokers) and change their attitudes toward tobacco-related issues through knowledge exchange. The main outcomes were the rate of information transfer, pattern of referrals, and attitudinal changes after participation.

## Methods

### Formative focus groups

To assist designing the content of the viral marketing campaign, we conducted four focus group sessions (each with 8 to 12 people) with 31 male and female Hong Kong residents aged 10 to 24. Eight of them (25.8%) self-reported being current smokers. During the sessions, the participants were invited to share their views and experiences concerning the factors that motivate young people to share information with friends and parents, those that motivate them to visit and revisit smoking cessation websites, the useful features and functions of a website promoting a smoke-free lifestyle, and the use of games on such a website.

About half of the participants suggested that an effective informative website should be attractive to web users on their first visit to encourage revisits. Those aged 10 to 19 reported that games and prizes would be helpful in encouraging users to revisit the site. They also indicated that the attractiveness of health promotion games did not depend on the game type. Instead, they perceived whether the game contained facts not commonly known to or shared among their peers was a more important motivating factor.

### Framework and design

Based on what we learnt from the focus groups, we decided to use an online multiple-choice quiz game competition as the content of the viral marketing campaign. We chose such game format over others because it poses minimal distractions, delivers information straightforwardly, and improves retention of the information delivered [31], [32].

The game website was displayed in traditional Chinese as our primary target was local Chinese youth. Every participant needed to register before participation. During the registration process, participants were asked to complete a short questionnaire on basic demographic information, baseline attitudes toward smoking, and referral codes (which they had received from those who referred them to the site). These referral codes were a set of 8-digit numbers automatically generated upon their registration and were unique to each user to track the propagation process.

The goal of the game competition from the participants' perspective was to obtain the highest score possible to become the champion of the campaign. Apart from learning correct smoking related knowledge, the champion will also receive an electronic device valued at US$650 for educational purpose. The participants could gain points to boost their scores in two ways. The first was to answer the online multiple-choice quiz questions. Smoking-related questions were presented on the quiz page one by one in random non-repeated order. Upon the completion of 20 quiz questions (one round), the users were given the chance to visit their member information panel for instantly updated scores. Each correct answer awarded 10 points to the user's account, but answering incorrectly or skipping a question did not result in any point deductions. Users were provided with immediate feedback on whether their answers were correct or wrong, which was found to be an effective learning strategy [33].

The quiz questions were designed based on the Elaboration Likelihood Model (ELM) of persuasion. The ELM asserts that there are two routes of persuasive message processing, namely the central path and the peripheral path [34]. The former requires greater mental effort but the attitude change resulted will have a more substantial effect, while the latter processing path require less mental effort but resulted in weaker short-term attitude change. The quiz questions were formed to enhance the likelihood to adopt the central path processing. To achieve such aim, three teenage volunteers were recruited and guided to form quiz questions that were concrete, specific and of personal relevance to ensure motivation and comprehensibility of adolescent participants [35], [36]. (An example question is: ‘What is the main reason of smoking among female smokers in Hong Kong according to the research of the University of Hong Kong?’) These characteristics, according to the ELM, will enhance the probability of choosing the central path and therefore ensure a stronger and more long-lasting effect [37]. The knowledge base of the quiz questions included international sources such as *Tobacco Atlas* and peer-reviewed journals and local sources such as the websites of the Hong Kong Government's Tobacco Control Office. An experienced clinician and a trained research assistant examined every set of 100 questions and discussed them with the question setters to ensure language accuracy and information reliability. This process was repeated 12 times for a total of 1,200 quiz questions. Finally, all of the questions were proofread, refined, and approved by the local Tobacco Control Office. Hyperlinks to these knowledge sources were presented in the campaign website so that participants who wanted to look for the correct answer could have an easy access.

The other way for participants to boost their scores was to make referrals. When making a referral, the participant needed to provide the person referred with the competition website address and his or her unique referral code. When the new participant input that code during registration, the system recognised the referral. The referrer then received both his or her own points from correct answers and all of the points from the correct answers of all direct referrals. Making a successful referral alone did not award any points to the referrers. The new participant also received his or her own referral code, which could be given to others when he or she subsequently made a referral. This point system was designed to encourage as many referrals as possible while minimizing the chance of making self-referring registration. A Facebook page dedicated to this campaign was also set up to enhance the knowledge exchange and communication among participants.

The participants were informed that the competition commenced on October 24, 2012 and ended on November 14, 2012. Participants were encouraged to answer an optional post-game questionnaire as part of the evaluation, and a US$2.6 (HK$20) cash voucher would be offered to those who did so as an incentive.

### Participants

We defined level-1 users as initial users who registered on the quiz game site without being referred to it by another user. Level-2 users were those who were referred to the game by a level-1 user, and level-3 users those who were referred to the game by a level-2 user, and so on. To recruit level-1 users to initiate the campaign, we conducted three 30-minute briefing sessions to a randomly selected class in grade 7 to 9 (totally three classes) in a local secondary school and posted banners in a university dorm and a non-governmental organization centre. The local secondary school was purposefully selected in a low socio-economic status district where its median monthly household income was ranked the lowest in Hong Kong [38]. Totally we recruited 121 level-1 users, of whom 112 were from the local secondary school, five from the university dorm, and four from the local non-governmental organization centre. Only two (1.7%) of the level-1 users identified themselves as smokers.

### Assessment

We evaluated the effectiveness of our viral marketing campaign by measuring three parameters. First, we measured the average referral rates of the sample, which was the average number of people a user referred to the game. Second, we compared the referral pathways originating from each level-1 age group (10 to 14, 15 to 19, and 20 to 24) to look for patterns of age influence. A referral pathway was the unidirectional network of users from a level-1 user to higher level users. Thus, a single pathway included a level-1 user and all downstream users (levels 2 to 6). Finally, we compared the participants' attitudes toward tobacco use and smoking before and after the competition using the Tobacco Attitude Survey, a 9-item inventory adapted from the Global Youth Tobacco Survey [39]. In the Tobacco Attitude Survey, 6 items were about general smoking-related perception and attitude, 2 were about perceived future smoking likelihood, 1 was about current smoking status. Participants were categorised in three groups according to their smoking status: (1) ‘non-smokers’ refer to those who have never smoked; (2) ‘ex-smokers’ refer to those who smoked previously (ranging from a few puffs to smoking every day) but stopped smoking already; (3) ‘current smokers’ refer to those who smoke tobacco sometimes or every day currently. The inventory was could be found in Text S1.

### Statistical analysis

The primary outcomes in this study were referral counts and attitudinal change. Referral counts were modelled using a zero-inflated negative binomial regression because of the excessive number of zeroes and over-dispersion shown in the empirical data [40]. Zero-inflated count models hypothesise that the outcome (i.e. the number of referrals in the present study) were generated from two separate processes: one for counts (count part) while the other for excess zeroes (zero-inflated part). The model's predictors included the participant's age, sex, and referral level. The participant groups that referred more users were identified by examining the coefficient estimates. To determine the referral pathways, we grouped all users into three groups based on the age group of the original level-1 users at the beginning of the pathways. Descriptive statistics on the age data were examined to identify any patterns or other notable observations.

Latent transition analysis (LTA) was used to assess any attitude shift among those who completed both pre- and post-survey [41], [42]. LTA is a longitudinal extension of the latent class analysis (LCA), which categorises subjects into several latent classes (categories that cannot be measured directly) according to their response to some items. LTA investigates the prevalence change of the latent classes along time and the likelihood to transit from one latent class into another (i.e. transition probabilities).

Six items related to general attitude toward smoking were entered into LTA to generate the latent classes. The number of latent classes was determined empirically using Akaike's Information Criterion (AIC), Bayesian Information Criterion (BIC), and entropy. AIC and BIC are comparative model fit indices where a lower number indicates a better model; entropy is an absolute indicator ranging from zero to one where a value close to 1 suggests a clearer class delineation. Item response probabilities of a LTA (or LCA) model are the probability of a person to choose an item conditional to his or her class membership, which provides an interpretation to the latent classes. Participants' attitudinal change as a whole was measured by the prevalence change among all three classes and the transition probabilities.

The changes in perceived smoking decision were analysed using two items in the Tobacco Attitude Survey: the perception of whether they would smoke with a close friend's offer and the perception of whether they would smoke in the coming 12 months. These two items were converted into binary variables in logistic regressions with ‘probably yes’ and ‘definitely yes’ treated as an event. The post-game binary variables were used as dependent variables in the models whereas the pre-game ones served as the offsets and potential contributing factors as independent variables.

### Ethics statement

Written informed consent was directly obtained from all participants before registration. Parents, caregivers and guardian of the participants aged below 18 were not asked to provide consent for their children as the study was of minimal risk and participants at the age of 10 and above should be fairly competent to consent this research [43], [44]. The study and the consent procedure were approved by the Institutional Review Board of the University of Hong Kong/Hospital Authority Hong Kong West Cluster.

## Results

### User statistics and referral rates

**Figure 1:**
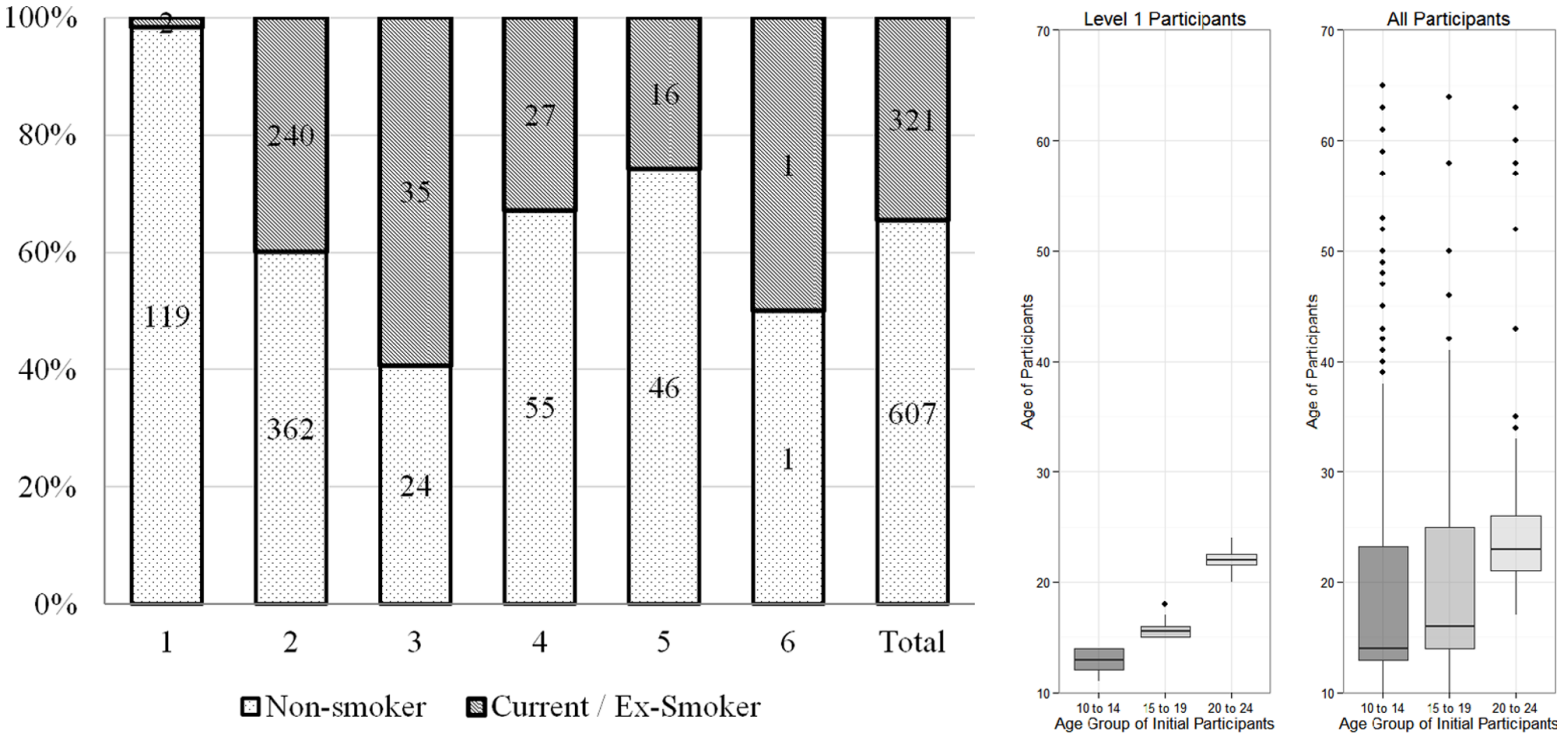
Referral process of the campaign. Left panel: number of non-smokers versus current or ex-smokers at each referral level. Right panel: Age distribution of all users in referral pathways originating from level-1 users in 10 to 14, 15 to 19, 20 to 24 age groups.

The online quiz game competition ran from October 24 to November 14, 2012. The competition began with 121 registered users, all of whom were aged 10 to 24. Of these 121 level-1 users, only two were ex-smokers and none currently smoked. By the end of the 22-day competition period, the competition had achieved six levels of referrals, including a total of 928 registered users of all ages (Figure 1), 205 (22.1%) of whom were ex-smokers and 116 (12.5%) of whom were current smokers. Of the 690 users aged 10 to 24 at the end of the competition period, 188 (27.2%) were either ex-smokers or current smokers. The average referral rate for all 928 registered users was 0.91. Ultimately, 655 of the 928 (70.6%) registered users actually answered the quiz questions or referred the game to other users, and 203 (21.9%) completed both the pre- and post-game questionnaire. A summary of the users' demographic characteristics is shown in Table 1.

**Table 1:** Demographics of the participants.

	All registered users	Users with successful referrals or answered quiz questions
	Number (Proportion)*	Number (Proportion)**
Sex		
Female	421 (0.45)	310 (0.47)
Male	507 (0.55)	345 (0.53)
Age		
10 to 14	343 (0.37)	248 (0.38)
15 to 19	173 (0.19)	131 (0.20)
20 to 24	174 (0.19)	128 (0.20)
25–39	180 (0.19)	103 (0.16)
40 or above	58 (0.06)	45 (0.07)
Smoking status		
Non-smokers	607 (0.65)	525(0.80)
Ex-smokers	205 (0.22)	89 (0.14)
Smokers	116 (0.13)	41(0.06)
Referral level		
1 (Seed users)	121 (0.13)	117 (0.18)
2–3	661 (0.71)	397 (0.60)
4–6	146 (0.16)	141(0.22)
Day of registration		
1–7	370 (0.40)	341 (0.52)
8–14	115 (0.12)	110 (0.17)
15–22	443 (0.48)	204 (0.31)
Number of quiz questions completed		
0	274 (0.30)	1 (0.00)
1–20	115 (0.12)	115 (0.18)
21–100	103 (0.11)	103 (0.16)
101–600	111 (0.12)	111 (0.17)
601–1200	325 (0.35)	325 (0.50)

*Proportion out of 928 registered users; ** proportion out of 655 users who answered quiz questions and/or made successful referrals. Note that the proportions may not add up to 1 owing to rounding error.

### Contributing factors of referrals

Table 2 shows the zero-inflated regression coefficients. The day of registration was positively associated with the likelihood of no referrals (OR  = 1.28, p<0.05), and the referral level was negatively associated with the number of successful referrals (RR  = 0.31, p<0.05). The zero-inflated component of the regression model showed that the older a user was and the later he or she registered, the more likely he or she made no referrals at all (OR  = 1.11, p<0.01). On the other hand, older users had higher referral numbers comparing to younger users (RR  = 1.24, p<0.05) as shown in the count part model.

**Table 2:** Factors associated with the number of referrals.

	Zero-inflated component			Count component		
Factors	Odds ratio (OR)	95% CI lower band	95% CI upper band	Relative ratio (RR)	95% CI lower band	95% CI upper band
Referral level	0.42	0.14	1.23	0.31*	0.12	0.82
Day of registration	1.28*	1.06	1.55	0.86	0.69	1.08
Age	1.11**	1.01	1.21	1.24*	1.08	1.43
Sex						
Female	1	-	-	1	-	-
Male	1.38	0.34	5.60	1.11	0.40	3.09
Attitude toward smoking in pre-game survey						
Negative	1	-	-	1	-	-
Neutral	7.13	0.79	64.32	1.63	0.55	4.82
Positive	8.89	0.65	122.36	2.84	0.37	21.86

*p-value <0.05; **p-value <0.01. Results estimated by zero-inflated negative binomial model using all 928 users who completed the registration process.

### Tracking of referral pathways

The referral patterns were discerned by tracking the referral pathways from the level-1 users to downstream users (Figure 1). By the end of the competition period, the 96 level-1 users aged 10 to 14 (median age  = 13) successfully referred the game to 476 users, (median age  = 16, age range  = 10 to 65), whereas the 18 level-1 users aged 15 to 19 (median age  = 15.5) referred the game to 144 users (median age  = 16, range  = 10 to 64) and the seven level-1 users aged 20 to 24 (median age  = 22) referred the game to 187 users (median age  = 23, range  = 17 to 63). Notably, the referral pathways originating with the level-1 users in the 10 to 19 age group reached all ages in the 22 days, but the same did not apply to level-1 users aged 20 to 24.

### Attitude shifts

The AIC and BIC were minimised in a 2- and 4-class LTA model respectively, while entropy was maximised in a 3-class model (Table S1). A 3-class model was thus chosen to balance all three criteria. We referred to these three classes hereafter as ‘positive’, ‘neutral’, and ‘negative’ to indicate participants' attitudes toward smoking based on the item response probabilities (Table 3). For instance, a young person in the ‘positive’ class had a favourable attitude toward smoking or cigarette use.

**Table 3:** Summary of LTA.

	Class 1 – Negative	Class 2 – Neutral	Class 3 – Positive
Class membership before game	0.57	0.17	0.26
Class membership after game	0.73	0.15	0.12
Transition probabilities after game			
To Class 1	0.91	0.48	0.52
To Class 2	0.07	0.19	0.18
To Class 3	0.02	0.34	0.30
Item response probabilities#			
Number of friends of boys as affected by smoking			
More	0.01	0.01	0.20
Indifferent	0.02	0.94	0.56
Less	0.98	0.05	0.23
Number of friends of girls as affected by smoking			
More	0.00	0.00	0.13
Indifferent	0.00	0.91	0.50
Less	1.00	0.09	0.37
Effect of smoking at party			
More comfortable	0.01	0.00	0.08
Indifferent	0.03	0.82	0.31
Less comfortable	0.97	0.18	0.61
Attractiveness of boys as affected by smoking			
More	0.00	0.00	0.11
Indifferent	0.00	0.97	0.12
Less	1.00	0.03	0.77
Attractiveness of girls as affected by smoking			
More	0.00	0.00	0.09
Indifferent	0.03	0.94	0.12
Less	1.00	0.06	0.79
Perceived harm to health			
Strongly disagree	0.01	0.02	0.13
Disagree	0.02	0.00	0.08
Agree	0.18	0.21	0.17
Strongly agree	0.79	0.78	0.62

# Item response probability is the probability to choose an item conditional on the persons' latent class membership. For example, in the question ‘number of friends of boys as affected by smoking’, a participant in Class 1 would have a 0.98 probability to select ‘Less’, 0.02 to select ‘Indifferent’ and 0.01 to select ‘More’. These probabilities help interpreting the latent classes and are conceptually similar to factor loadings in factor analysis. For detailed technical specifications, please refer to Lanza *et al* and Rindskopf [41], [42].

LTA showed a marked increase in the proportion of participants holding negative attitudes toward smoking for 57% before to 73% after the competition. The percentage of those holding positive attitudes toward smoking fell from 26% to 12% (Table 3). The odds ratio of transitioning from a neutral to a negative attitude after completing one round of the game (20 quiz questions) was 1.003, whereas that of transitioning from a positive to a negative attitude after each round was 1.004. This means that the more quiz questions the users attempted, the more likely they became negative towards smoking.

Logistic regressions have revealed that the number of quiz questions completed during the campaign was the only statistically significant predictor for perceived smoking decision after adjusting for potential confounders and pre-game perceived decision. Odds ratios were 0.96 (p<0.05) and 0.95 (p<0.01) for the perceived smoking decision with a close friend's offer and for the perceived smoking decision in the coming 12 months respectively, indicating that the more quiz questions the users attempted, the less likely they perceive their chance of smoking in the future.

## Discussion

To the best of our knowledge, this study is the first to apply viral marketing specifically to promote smoke-free attitude. Our campaign showed promising results in terms of participants' attitudinal change toward smoking and the ability in reaching a large group of youths, including smokers, within a short period of time.

Although the short study duration did not allow us to observe participants' change in smoking habit, the significant improvement in participants' attitude against smoking and the decrease in their intention to smoke (e.g. perceived decision of smoking with a close friend's offer) are also valuable and favourable outcomes. Undoubtedly, the change in attitude and intention may not necessarily lead to smoking cessation or prevention, but these are likely to influence future smoking related behaviour according to the Theory of Planned Behaviour and previous empirical studies [45]–[47]. In addition, utilising the Elaboration Likelihood Model of persuasion in quiz question setting may prolong the effect of attitudinal shift and therefore increase the depth of impact. All these evidences suggest that the present campaign could have a potential contribution to tobacco control.

Apart from promoting a smoke-free lifestyle, the present study also demonstrates how ‘Web 2.0’ (emphasising the interactions among users) could successfully engage participants in a health promotion context. Previous health promotion or education game models were primarily based on knowledge transfer and instant information flow [48]. With careful planning and implementation, such approach was successful in attracting the targets and transferred the correct knowledge to the target audience but sustaining engagement seemed to be difficult. An example for such obstacle could be found in the Reach Out Central, a serious game to enhance youth's mental well-being [48]. Comparing to the Reach Out Central, the present study seems to be more successful in engaging youths. Such merit could be attributed to the additional component of this campaign over Reach Out Central, i.e. the social interaction based on the user referral system. Yet further investigation is needed to understand the contributing factors for successful and long-term engagement for Internet health intervention models.

Research has illustrated viral marketing to have additional benefits over traditional advertising such as TV commercials [49], [50]. In general, viral marketing campaigns appear to be more cost-effective and can reach more people effectively [49]. Unlike traditional marketing schemes, the person-to-person information exchange process in viral marketing can be precisely tracked by collaborating with social networking sites or participants, or by hosting a web application, as we did in the present study [50].

Within the short study period of 22 days, there was an almost sevenfold increase in registered users with 6 referral levels. Although this is an encouraging result, the actual potential of viral marketing may not have been fully reflected. A previous viral marketing campaign study, for example, was able to reach 110,200 web users in 15 days starting from just 215 level-1 users [27]. Better understanding of the factors that influence referral rates across campaigns could help to plan for a better implementation in the future.

Interestingly, although an older user in this study was less likely to make a referral attempt, he or she was more likely to succeed in that attempt than a younger user. A possible reason is that older users are more receptive to anti-smoking messages [51], though the overall mechanism remains unknown and merits further research.

The tracking of referral pathways revealed that users in all three level-1 age groups tended to pass on information about the game to others close to them in age. Those level-1 users in the 10 to 19 age group successfully spread the website to users of all ages which suggests that this age group is particularly socially connected online. Such connectedness appears less prevalent among level-1 users aged 20 to 24, who spread the website to their peers of similar age and older individuals but not to those in the younger age group. This is one of the first observations of such an interesting phenomenon. It is possible that users of an older age group did not attempt to refer younger social contacts or their social network consists of fewer younger friends. Such finding provides some knowledge on the choice of campaign starters in future peer-referring health interventions: adolescents, rather than young adults, should be chosen as campaign starters if the message is targeting on all ages.

Although this study provides some insight into online social interactions of young people, the roles that social influence and other factors played in our campaign remain elusive. De Bruyn and Lilien found demographic similarities between a referrer and recipient to be significantly negatively associated with the latter's likelihood of opening the former's e-mail message, taking an interest in the information therein, and adopting the product [52]. Their finding appears to contradict our finding that the quiz game participants tended to successfully refer others of a similar age. However, De Bruyn and Lilien did note that demographic similarity's effect on information exchange is dependent on the situation, and other sociological studies have produced mixed results in this regard [53]–[55]. They also proposed that when information exchange requires a high degree of trust, people tend to exchange information with demographically similar others, which suggests that a high degree of trust may be involved when young people exchange tobacco-related information. As a result, the peer-driven referrals in Internet viral marketing may be a better model for anti-smoking education than the conventional health promotion model.

Last but not least, a significant amount of smokers were reached and referred from a predominantly non-smoker level-1 users in the present study, which actually could be a key for future risk behaviour interventions among youth. Teenage smoking has become a serious public health concern for decades, but most intervention programmes were not successful to reach youth smokers [56]. One reason is that youth smoking is usually considered socially unacceptable and thus most teenage smokers are not ready to be approached by healthcare providers. Nevertheless, through the peer-referral processes in the present campaign, these barriers seem to be softened. The ability to reach risk-taking youths makes the viral marketing model a promising entry point for interventions. Hence, similar models should be considered for other risk-taking behaviours, like substance abuse and alcoholism.

### Limitations

In spite of the efforts to ensure validity, there were still limitations in our study as in most similar research [57]. First, the relatively short study duration did not allow us to directly observe participants' behaviour changes, or to examine the sustainability of the observed attitudinal changes. Second, the relatively low response rate (21.9%) for the post-game survey may lead to a potential bias in the evaluation: respondents to the post-game survey may be more enthusiastic to the game than the non-respondents, which may account for a more positive attitude change. We should not, however, underestimate the potential of the campaign effectiveness as attempting quiz questions was found negatively associated with perceived smoking decisions as shown in Table 4. Third, we could not eliminate the possibility for self-referral, even though it should be unlikely. Our system did not allow users to create accounts with an existing e-mail address or telephone number, but they may still register with multiple e-mail accounts and telephone numbers. We believe the problem is unlikely to influence the overall conclusions of the present study as the incentive of making self-referral was minimised – making a successful referral alone did not constitute extra points to the referrers. Last but not least, it should be noted that the sample in this study has a lower female-to-male ratio when compared to the overall local figure [3]. Such phenomenon could be due to the gender difference in Internet use and/or digital literacy [58]. Thus, effectiveness of similar campaign may not be as effective in areas with lower digital and Internet literacy.

**Table 4:** Factors associated with the change in perceived smoking decision.

	Perceived smoking decision with a close friend's offer (‘Probably yes’ and ‘Definitely yes’ as event)			Perceived smoking decision in the coming 12 months (‘Probably yes’ and ‘Definitely yes’ as event)		
Factors	Odds ratio (OR)	95% CI lower band	95% CI upper band	Odds ratio (OR)	95% CI lower band	95% CI upper band
Age	1.04	0.99	1.10	1.04	0.99	1.10
Sex						
Female	1	-	-	1	-	-
Male	3.13	0.74	13.29	3.13	0.74	13.29
Number of referrals	0.77	0.21	2.84	0.52	0.09	3.01
Quiz questions completed (every 20 questions)	0.96*	0.93	0.99	0.95**	0.92	0.98
Quiz questions correct answer rate	238.29	0.27	>1000	1.61	0.01	174.46
Attitude toward smoking in pre-game survey						
Negative	1	-	-	1	-	-
Neutral	0.00	0.00	>1000	0.00	0.00	>1000
Positive	2.00	0.52	7.65	0.64	0.18	2.29

*p-value <0.05; **p-value <0.01. Results estimated by logistic regression model using 204 users who has completed both the pre- and post-game surveys.

## Conclusions

In conclusion, this study provides an important piece of information about young people's online social interactions in the health promotion context in the most urbanised and westernised city of China. We have also shown that an online quiz game competition is an effective way of disseminating tobacco-related health information. Most importantly, we have demonstrated that Internet viral marketing possesses enormous potential for health education, which is still underutilised currently.

## Supporting Information

Questions S1 **Sample quiz questions of the intervention.** A sample set of 20 quiz questions translated from the original Chinese question bank.
(XLSX)

Table S1 **Selection criteria for latent transition models.** AIC: Akaike's Information Criterion; BIC: Bayesian Information Criterion; df: degrees of freedom. Fit indices for the latent class models on attitudes toward cigarette use. Sample is all 203 users who completed both the pre- and post-intervention questionnaire.
(DOCX)

Text S1 **Tobacco Attitude Survey.** Adapted from Global Youth Tobacco Survey, this inventory was administered before and after the intervention to measure participants' smoking status, intention to smoke, and attitude towards smoking.
(DOCX)
