# Supplementary material for: Use of Internet Viral Marketing to Promote Smoke-Free Lifestyles among Chinese Adolescents
Source: PLoS One. 2014 Jun 9;9(6):e99082. doi: 10.1371/journal.pone.0099082 (PMC4049615; doi:10.1371/journal.pone.0099082)
Supplement: Table S1 — Selection criteria for latent transition models. AIC: Akaike's Information Criterion; BIC: Bayesian Information Criterion; df: degrees of freedom. Fit indices for the latent class models on attitudes toward cigarette use. Sample is all 203 users who completed both the pre- and post-intervention questionnaire. (DOCX) [file pone.0099082.s002.docx]

# Table S1: Selection criteria for latent transition models

| No. of  latent classes | Log-Likelihood | AIC | BIC | Entropy | df |
| --- | --- | --- | --- | --- | --- |
| 2 | -605.05 | 280.68 | 370.14 | 0.95 | 944 |
| 3 | -578.05 | 254.68 | 390.52 | 0.96 | 930 |
| 4 | -561.12 | 138.83 | 431.06 | 0.93 | 916 |
| 5 | -543.43 | 241.45 | 470.06 | 0.92 | 902 |
| 6 | -534.07 | 250.72 | 525.71 | 0.95 | 888 |

AIC: Akaike’s Information Criterion; BIC: Bayesian Information Criterion; df: degrees of freedom. Fit indices for the latent class models on attitudes toward cigarette use. Sample is all 203 users who completed both the pre- and post-intervention questionnaire.

# 
