# Supplementary material for: Use of Internet Viral Marketing to Promote Smoke-Free Lifestyles among Chinese Adolescents
Source: PLoS One. 2014 Jun 9;9(6):e99082. doi: 10.1371/journal.pone.0099082 (PMC4049615; doi:10.1371/journal.pone.0099082)
Supplement: Text S1 — Tobacco Attitude Survey. Adapted from Global Youth Tobacco Survey, this inventory was administered before and after the intervention to measure participants' smoking status, intention to smoke, and attitude towards smoking. (DOCX) [file pone.0099082.s003.docx]

# Text S1: Tobacco Attitude Survey (Adapted from Global Youth Tobacco Survey)

1. Do you think boys who smoke tobacco have more or fewer friends?

① More friends ② Fewer friends ③ No difference from non-smokers

1. Do you think girls who smoke tobacco have more or fewer friends?

① More friends ② Fewer friends ③ No difference from non-smokers

1. Do you think smoking tobacco helps people feel more comfortable or less comfortable at celebrations, parties, or in other social gatherings?

① More comfortable ② Less comfortable ③ No difference from non-smokers

1. Do you think smoking tobacco makes boys more or less attractive?

① More attractive ② Less attractive ③ No difference from non-smokers

1. Do you think smoking tobacco makes girls more attractive or less attractive?

① More attractive ② Less attractive ③ No difference from non-smokers

1. Do you think smoking tobacco is harmful to your health?

① Definitely not ② Probably not ③ Probably yes ④ Definitely yes

1. If one of your close friends offered you a cigarette, would you smoke it?

① Definitely not ② Probably not ③ Probably yes ④ Definitely yes

1. At any time during the next 12 months do you think you will smoke a cigarette?

① Definitely not ② Probably not ③ Probably yes ④ Definitely yes

1. Please read the following sentences, then choose the most appropriate (please choose only one answer):

① I have never smoked tobacco.

② I have only smoked tobacco once or a few times (including If you were just trying or experimenting).

③ I used to smoke tobacco in the past (not every day), but I don’t smoke now.

④ I used to smoke tobacco every day in the past, but I don’t smoke now.

⑤ I smoke tobacco sometimes (not every day).

⑥ I smoke tobacco every day.
